# TAP1-Deficiency Does Not Alter Atherosclerosis Development in *Apoe* ^−/−^ Mice

**DOI:** 10.1371/journal.pone.0033932

**Published:** 2012-03-30

**Authors:** Daniel Kolbus, Irena Ljungcrantz, Ingrid Söderberg, Ragnar Alm, Harry Björkbacka, Jan Nilsson, Gunilla Nordin Fredrikson

**Affiliations:** 1 Department of Clinical Sciences, Skane University Hospital Malmö, Lund University, Malmö, Sweden; 2 Faculty of Health and Society, Malmö University, Malmö, Sweden; King's College London, University of London, United Kingdom

## Abstract

Antigen presenting cells (APC) have the ability to present both extra-cellular and intra-cellular antigens via MHC class I molecules to CD8^+^ T cells. The cross presentation of extra-cellular antigens is reduced in mice with deficient Antigen Peptide Transporter 1 (TAP1)-dependent MHC class I antigen presentation, and these mice are characterized by a diminished CD8^+^ T cell population. We have recently reported an increased activation of CD8^+^ T cells in hypercholesterolemic *Apoe^−/−^* mice. Therefore, this study included TAP1-deficient *Apoe^−/−^* mice (*Apoe^−/−^Tap1^−/−^*) to test the atherogenicity of CD8^+^ T cells and TAP1-dependent cross presentation in a hypercholesterolemic environment. As expected the CD8^+^ T cell numbers were low in *Apoe^−/−^Tap1^−/−^* mice in comparison to *Apoe^−/−^* mice, constituting ∼1% of the lymphocyte population. In spite of this there were no differences in the extent of atherosclerosis as assessed by *en face* Oil Red O staining of the aorta and cross-sections of the aortic root between *Apoe^−/−^Tap1^−/−^* and *Apoe^−/−^* mice. Moreover, no differences were detected in lesion infiltration of macrophages or CD3^+^ T cells in *Apoe^−/−^Tap1^−/−^* compared to *Apoe^−/−^* mice. The CD3^+^CD4^+^ T cell fraction was increased in *Apoe^−/−^Tap1^−/−^* mice, suggesting a compensation for the decreased CD8^+^ T cell population. Interestingly, the fraction of CD8^+^ effector memory T cells was increased but this appeared to have little impact on the atherosclerosis development.

In conclusion, *Apoe^−/−^Tap1^−/−^* mice develop atherosclerosis equal to *Apoe^−/−^* mice, indicating a minor role for CD8^+^ T cells and TAP1-dependent antigen presentation in the disease process.

## Introduction

According to the current view, oxidized LDL (oxLDL) within the vascular wall initiates an immune response leading to influx of phagocytes and T cells to the inflammatory site, which ultimately leads to a chronic inflammation and growth of atherosclerotic lesions [1], [2]. Hypercholesterolemia promotes atherosclerosis and leads to an imbalance in pro- and anti-atherogenic T cell populations. Pro-atherogenic T cells specific for oxLDL are found in atherosclerotic lesions and circulation [3], [4] and mice deficient in CD4^+^ T cells displayed decreased lesion size [5], [6]. In contrast, Elhage *et al.* reported increased lesion development in *cd4^−/−^* mice [7] whereas Dansky *et al.* stated that T and B cells had a minor impact in atherosclerosis [8]. The balance between pro- and anti-atherogenic signals within the T cell population may determine disease development. The induction of regulatory CD4^+^ T cells is anti-atherogenic [9] while depletion leads to increased lesion development [10], highlighting the dual role of CD4^+^ T cells and that imbalance may accelerate atherosclerosis. Less focus has been on the role of CD8^+^ T cells in atherosclerosis. Elhage *et al.* reported that aortic lesions of *Apoe^−/−^* mice deficient in CD8^+^ T cells receiving chow diet did not differ in size compared to *Apoe^−/−^* mice with a normal CD8^+^ T cell population [7]. In contrast CD8^+^ T cells were found to comprise up to 50% of the lymphocyte population in advanced human atherosclerotic lesions [11] and *Apoe^−/−^* mice expressing β-galactosidase (β-gal) in aortic smooth muscle cells (SMCs) developed CD8^+^ T cell driven arteritis and atherosclerosis upon immunization with β-gal specific dendritic cells (DC) [12]. We recently conducted a study in hypercholesterolemic *Apoe^−/−^* mice comparing the CD4^+^ and CD8^+^ T cell responses early in the disease process. There was an induction of CD8^+^IFN-γ^+^ cells in heart draining lymph nodes and spleen and increased proliferation of CD8^+^ splenocytes compared to CD4^+^ counterparts [13]. This prompted us to formulate the hypothesis that a hyperlipidemic diet leads to activation of effector CD8^+^ T cells that drive development of atherosclerotic plaques in a CD4^+^-independent way. To test this, *Tap1^−/−^* mice [14] were crossed with *Apoe^−/−^* mice to generate a hypercholesterolemic mouse model with low CD8^+^ T cell numbers. The antigen peptide transporter 1 (TAP1) together with TAP2 constitutes a MHC class I transporter maintaining the major pathway of MHC class I surface expression. TAP1 is important for thymic differentiation of T cells and deficiency result in a diminished pool of peripheral CD8^+^ T cells [15]. Since CD8^+^ T cells can get activated by extra-cellular antigens presented via MHC class I cross presentation [16], diminishing this pathway would possibly affect pro-atherogenic CD8^+^ T cell responses. Surprisingly, the *Apoe^−/−^Tap1^−/−^* mice developed lesions of the same size as the *Apoe^−/−^* mice, indicating that TAP1-deficiency has no or minor effect on atherosclerosis.

## Materials and Methods

### Ethics Statement

The Local Animal Care and Use Committee at Lund University approved (Permit numbers M159-07 and M153-10) the experimental protocol used in the study. All surgery was performed under anesthesia, and all efforts were made to minimize suffering.

### Animals

Female apolipoprotein E deficient- and TAP1-deficient mice on a C57BL/6 background were purchased from Jackson Laboratories, USA and C57BL/6 (wild type, WT) mice were obtained from in house breeding. To generate double deficient mice, *Apoe* ^−/−^ and *Tap1* ^−/−^ mice were crossed to obtain parental genotypes. The number of animals used was 10–13 and 7–10 per experimental group for mice sacrificed at 26 and 14 weeks of age, respectively. All animals were controlled for respective genetical background by PCR assay. *Tap1* ^−/−^ and *Apoe* ^−/−^ *Tap1* ^−/−^ mice did not have detectable levels of *Tap1*. The animals were kept under controlled laboratory conditions in individually ventilated cages (IVC) and food and water were provided *ad libitum*. The *Apoe^−/−^Tap1^−/−^* mice did not display any obvious phenotypically differences in comparison to *Apoe^−/−^* mice such as body weight and blood lipids (table 1 and 2). Further, we recorded no premature death or abnormal behavior. Thus, they appeared healthy, but *Tap1* ^−/−^ mice had bald, non-fur covered areas in the back, which was not apparent in *Apoe* ^−/−^ *Tap1* ^−/−^, *Apoe* ^−/−^ or WT mice. At the age of 6 weeks, diet was shifted from chow to high fat diet (HFD; 0.15% cholesterol and 21% fat (Lantmännen, Sweden)). The mice were sacrificed at 8 or 20 weeks after diet change by intraperitoneal injection of Xylazine (Rompun, Bayer Health care), and Ketamine (Ketalar, Pfizer), in sodium chloride solution (0.9%) and exsanguinated by cardiac puncture, perfused with 5 ml PBS (pH 7.4), followed by removal of spleen and lymph nodes and thereafter infusion of 5 ml of the fixative Histochoice (Amresco, Solon, Ohio). The aorta was then dissected free of connective tissue and fat, cut longitudinally, mounted *en face* and stored in Histochoice. The heart was collected and stored in Histochoice at 4°C until processing. Plasma was collected from cardiac punction and stored at −80°C until assayed. In a second set of experiments female mice (6 *Apoe* ^−/−^ mice and 6 *Apoe* ^−/−^ *Tap1* ^−/−^ mice) were sacrificed 22 weeks after diet change. However, histochoice was not used, lymphoid organs were not assayed and the heart was snap frozen in liquid nitrogen and stored at −80°C.

**Table 1:** Lesion size, weight, plasma cholesterol and triglycerides at 14 weeks of age.

	WT	*Tap1^−/−^*	*Apoe^−/−^*	*Apoe^−/−^Tap1^−/−^*
**Lesion size (mm^2^×10^−3^)**	n/a	n/a	318.7±93.9	350.1±119.8
**Weight (g)**	25.5±4.4	20.6±1.4^a^	22.9±2.4	21.3±1.9
**Cholesterol (mg/dl)**	86.8±26.8	71.9±15.3	604.6±87.3^b^	650.0±92.7^b^
**Triglycerides (mg/dl)**	25.7±15.7	24.7±7.1	42.6±5.4^c^	50.0±7.9^d^

n/a, not applicable;

a *P*<0.01 vs. WT,

b *P*<0.001 vs. WT and *Tap1^−/−^*,

c *P*<0.01 vs. *Tap1^−/−^*,

d *P*<0.001 vs. WT and *Tap1^−/−^*. The number of animals in respective group was 7 (WT), 10 (*Tap1^−/−^*), 10 (*Apoe^−/−^*) and 7 (*Apoe^−/−^Tap1^−/−^*). In the triglyceride assay, one *Apoe^−/−^Tap1^−/−^* mouse displayed 3 times higher values than group mean and was excluded from analysis.

**Table 2:** Plasma cholesterol, triglycerides and weight at 26 weeks of age.

	WT	*Tap1^−/−^*	*Apoe^−/−^*	*Apoe^−/−^Tap1^−/−^*
**Cholesterol**	104.3±21.0	85.1±11.6	792.6±101.3^a^^,^ b	740.1±134.6^a^^,^ b
**Triglycerides**	24.5±18.8	33.8±23.8	47.6±17.7^c^	54.0±20.1^d^^,^ e
**Weight**	33.5±6.6^f^	22.2±3.6	24.4±3.1	22.5±1.6

a *P*<0.01 vs. WT,

b *P*<0.001 vs. *Tap1^−/−^*,

c *P*<0.01 vs. WT,

d *P*<0.001 vs. WT,

e *P*<0.05 vs. *Tap1^−/−^*,

f *P*<0.001 vs. *Tap1^−/−^*, *Apoe^−/−^* and *Apoe^−/−^Tap1^−/−^*. The number of animals in respective group was 12 (WT), 13 (*Tap1^−/−^*), 11 (*Apoe^−/−^*) and 13 (*Apoe^−/−^Tap1^−/−^*).

### Analysis of plaque macrophage, T cell and neutral lipid content

The heart was embedded in OCT (Optimal Cutting Temperature; Tissue-TekZoeterwoulde, The Netherlands) and frozen sections of 10 µm were collected. All staining experiments were done at room temperature if not stated otherwise. Macrophage stained sections were fixed in ice-cold acetone for 10 minutes, incubated with 0.5% Triton-×100 (Merck Chemicals, Darmstadt, Germany) and 3% H_2_O_2_ (Apoteket AB, Sweden) for 5 minutes, each step separated by PBS rinsing. Thereafter the sections were blocked with 10% mouse serum in PBS for 30 minutes and quickly dipped in PBS before staining with a rat anti-mouse MOMA-2 antibody (monocyte/macrophage, BMA Biomedicals, Augst, Switzerland) diluted in 10% rat serum in PBS and incubated at +4°C over night. The slides were incubated with biotinylated rabbit anti-rat IgG (BA-4001, Vector Laboratories, Burlingame, CA) for 50 minutes followed by a 30-minute incubation with ABC solition (ABC elite, Vector Laboratories) and color development using the DAB detection kit (Vector Laboratories). For T cell detection, slides were fixed in acetone, washed and permeabilized using Triton-×100 followed by incubation with H_2_O_2_ according to the MOMA-2 protocol. Slides were incubated for 30 minutes in 10% goat serum followed by incubation with rabbit anti-human CD3 (cross-reacts with mouse CD3 [17]; A0452, Dako Cytomation, Fort Collins, CO) diluted in 2% goat serum at 4°C overnight. This was followed by a 50-minute incubation with biotinylated goat anti-rabbit IgG (BA-1000, Vector Laboratories) and the ABC/DAB procedure according to the MOMA-2 protocol. Omissions of the primary or secondary antibodies were used as negative controls. To analyze T cell subsets in aortic root lesions sections of snap frozen heart tissue were fixed in acetone for 15 minutes, rehydrated in PBS for 10 minutes and incubated with 0.3% H_2_O_2_ for 2 minutes followed by PBS wash. The sections were blocked with 10% mouse serum for 30 minutes followed by a 15 minute block with avidin (Thermo Scientific) and a 15 minute block with biotin (Thermo Scientific). The slides were washed with PBS and incubated with rat anti-mouse CD4 (553044, clone RM4-5, BD Pharmingen) or rat anti-mouse CD8a (553029, clone 53-6.7, BD Pharmingen) diluted in 10% mouse serum for 30 minutes. Omission of the antibody was used as negative control. The color development procedure was as reported for the MOMA-2 protocol. For lipid staining, the sections were put in 0.24% Oil red O diluted in 60% isopropanol for 10 minutes, rinsed with tap water to remove redundant Oil red O, and dipped in 60% isopropanol followed by washing with dH_2_O before nuclear staining with hematoxylin for 15 seconds. *En face* preparations of the aorta were washed in distilled water, dipped in 78% methanol, and stained for 40 minutes in 0.16% Oil red O dissolved in 78% methanol/0.2 mol/L NaOH as previously described [18]. The cover slides were mounted with a water soluble mounting media L-550A (Histolab, Göteborg, Sweden). Oil red O stains lipids red, which makes the plaques bordeaux colored. Stained area and total lesion area of all immunohistochemical and histochemical analyses were quantified by microscopy and computer aided morphometry by a blinded observer (BioPix IQ 2.0, Göteborg, Sweden).

### Cell preparation and flow cytometry

#### A. Mice analyzed at 26 weeks of age

The spleen and mediastinal lymph nodes (MeLN) were meshed through a cell strainer (70 µm, BD Bioscience). The single cell suspension of the lymph nodes were washed in RPMI medium (Gibco, USA) and resuspended in complete medium (RPMI 1640 supplemented with 10% FCS, 1% Sodium pyruvate, 1% Hepes, 1% Penicillin/Streptomycin, 1% L-Glutamine and 0.1% β mercaptoethanol [Gibco]). Splenocytes were pelleted and resuspended in red blood cell lyzing buffer (Sigma) for two minutes at room temperature to remove erythrocytes. Cells were washed and resuspended in complete medium. Spleen and MeLN cells were distributed in 96-well round bottom plates (Sarstedt, Landskrona, Sweden) at a density of 1.5×10^6^ cells/ml for antibody staining. ^Ce^lls were then divided into groups being antibody stained directly (day 0) or after ConcanavallinA (ConA) incubation (day 2). Directly stained cells were incubated with a Fc-receptor blocking antibody (FcR;CD16/32; clone 93, Biolegend) for 5 minutes followed by incubation with either of two antibody panels. For panel one; CD80-PE (clone 16-10A1), CD11c-PE/Cy7 (N4-18), CD86-PB (GL-1) and I-A/I-E-A700 (M5-114.15.2) (all Biolegend, San Diego, CA, USA except CD80-PE; eBioscience, San Diego, CA, USA) and panel two CD44-AF488 (IM7), CD122-PE (5H4), CD3-PE/Cy7 (145-2C11), CD4-PB (GK1.5), CD62L-APC (MEL-14) and CD8-APC/Cy7 (53-6.7) at 4°C for 30 minutes. 7AAD (Sigma; 1 µl/sample) was included in both panels. Further, cells were washed with FC buffer (0.5% bovine serum albumin (Sigma) and 0.5 mM EDTA in phosphate buffered saline) and incubated with 4% PFA (paraformaldehyde; Sigma) for 20 minutes followed by wash and resuspension in FC buffer. Cells in the day 2-group were first incubated in presence or absence of 0.625 µg/ml ConA (Sigma) and incubated for 2 days at 37°C and 5% CO_2_. The antibody staining procedure was identical to the procedure for the day 0-group and both groups were acquired in the same flow cytometry run. Measurements were performed using a Cyan ADP (Beckman Coulter, Brea, CA, USA), analyses were performed using FlowJo (Treestar inc., Ashland, OR, USA, version 7.6.1) and gating was adjusted using fluorescence minus one (FMO) control staining.

#### B. Mice analyzed at 14 weeks of age

The MeLN were meshed through a cell strainer (70 µm, BD Bioscience). The single cell suspension of the lymph nodes was washed in cell medium (PBS (Gibco), 2% FCS, 2 mM EDTA) and resuspended in RPMI-1640 (Gibco). The spleens were minced into pieces and incubated in spleen dissociation buffer (Stemcell Technologies, Vancouver, Canada) according to manufacturer's instructions. Cells were resuspended in RPMI-1640 and distributed in 96-well round bottom plates (Sarstedt) at a density of 1×10^6^ cells/ml. Thereafter cells were incubated with a Fc-receptor blocking antibody (FcR;CD16/32; clone 93, Biolegend) for 5 minutes followed by incubation with CD3-PE/Cy7 (145-2C11), CD4-PB (GK1.5) and CD25-APC (PC61; all Biolegend) for 30 minutes at 4°C. The cells were resuspended in Fix/Perm solution (eBioscience), washed with permeabilization buffer (eBioscience) and blocked with FcR for 5 minutes prior to incubation with FoxP3-PE (MF-14, Biolegend) for 30 minutes at 4°C. Cells were washed with permeabilization buffer, resuspended in FC buffer (1% fetal calf serum (Gibco) and 0.5 mM EDTA in PBS) and acquired as described for the 26 weeks old mice.

### Proliferation of splenocytes from mice at 14 weeks of age

Spleen CD11c^+^ cells were isolated using Easy sep CD11c^+^ positive selection kit (Stemcell Technologies) with a purity of 71%. Subsequently CD4^+^ cells were isolated using Easy sep CD4c^+^ positive selection kit (Stemcell Technologies) with a purity of 70% followed by CD8^+^ cell isolation using Easy sep mouse CD8^+^ T cell enrichment kit (Stemcell Technologies) with a purity of 89%. CD11c^+^, CD4^+^ and CD8^+^ cells (each 5×10^4^/well) were cultured in medium (RPMI-1640 medium containing 10% heat-inactivated fetal calf serum, 1 mmol/L sodium pyruvate, 10 mmol/L Hepes, 50 U penicillin, 50 µg/mL streptomycin, 0.05 mmol/L β-mercaptoethanol and 2 mmol/L L-glutamine, Gibco, Invitrogen) in 96-well round bottom plates (Sarstedt) in presence or absence of 0.625 µg/ml ConA (Sigma) for 72 hours at 37°C and 5% CO_2_. To measure cell proliferation, [methyl-^3^H] thymidine (Perkin-Elmer, Waltham, MA, USA) was added (20 µl/well diluted 1∶20) and incubated 16–20 hours. Macromolecular material was then harvested on glass fiber filters using a Printed Filtermat A (1450-421, Wallac Oy, Turku, Finland). Filters were air-dried and the bound radioactivity was measured in a beta-counter (Wallac 1450 MicroBeta, Ramsey, MN, USA).

### Analysis of cytokines in plasma

Cytokine concentrations in plasma were analyzed using a mouse Th1/Th2 9-Plex (IFN-γ, IL-1β, TNF-α, IL-2, IL-12, IL-4, IL-5, IL-10 and KC/GRO/CINC (CXCL1) Ultra-Sensitive Kit (Meso Scale Discovery, Gaithersburg, MO, USA), following the instructions of the manufacturer. Samples were analyzed separately for each individual animal. The lower detection limit in this assay was 1.02–9.50 pg/ml depending on the cytokine assayed.

### Analysis of cholesterol and triglyceride content

Total plasma cholesterol and plasma triglycerides were quantified with colorimetric assays, Infinity™ Cholesterol and Triglyceride (Thermo Electron, Waltham, MA, USA).

### Statistical analysis

Analysis of data was performed using unpaired *t* test or Mann Whitney test for skewed data. Data are presented as mean±standard deviation. Kruskal-Wallis test and Dunn's post hoc test was used to analyze plasma triglyceride data. Analysis was performed using GraphPad Prism 5.01 (Graphpad software, La Jolla, CA, USA) and a level of *P*<0.05 was considered significant.

## Results

### Characterization of the mouse model and quantification of atherosclerosis

**Figure 1:**
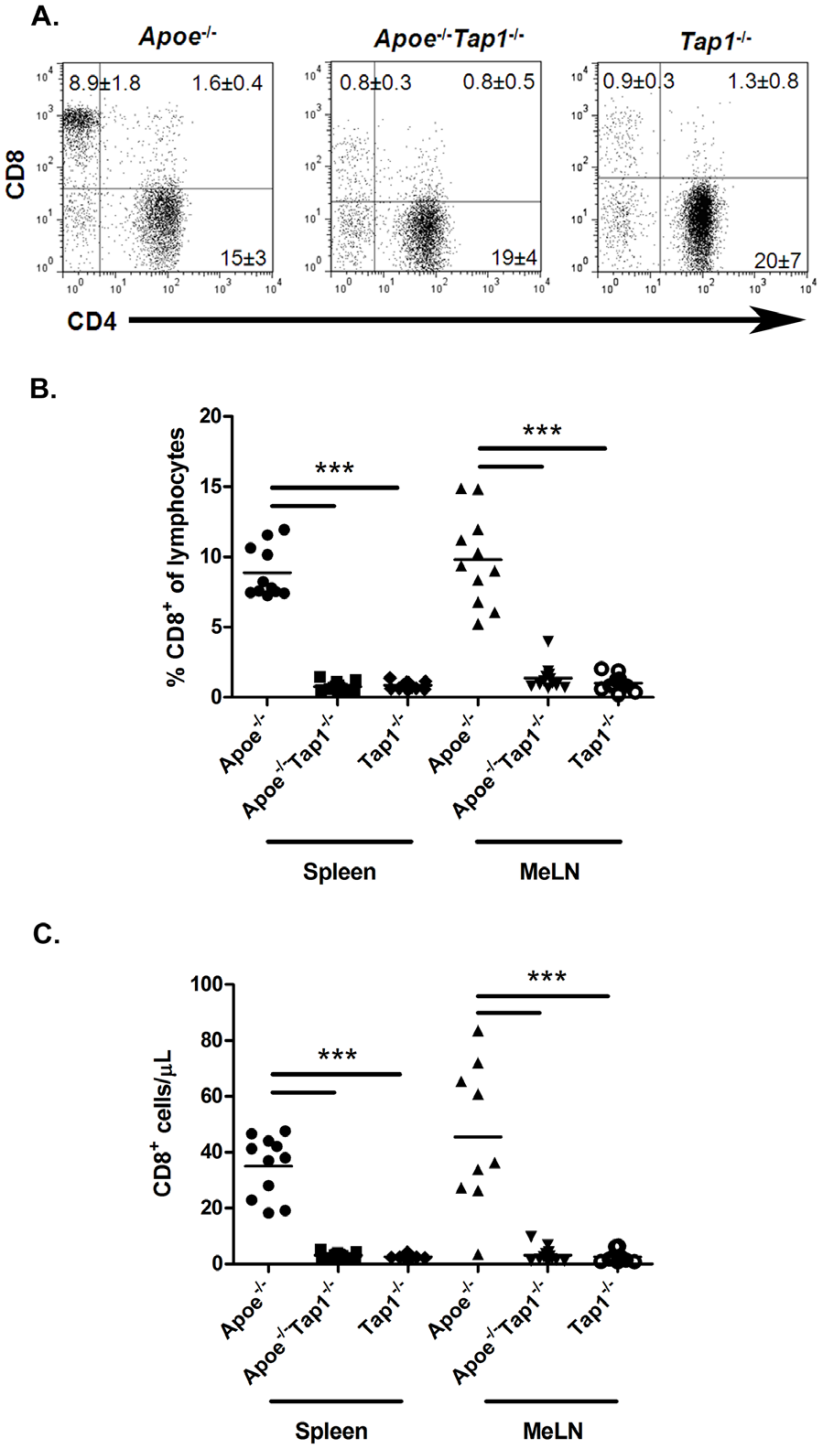
CD8^+^ T cells in spleen and mediastinal lymph nodes (MeLN). Flow cytometry graphs showing the cell populations in spleen from one representative mouse from each group (A). Numbers given in the graphs are per cent cells out of all lymphocytes. The fraction (B) and total cell count (C) of CD8^+^ T cells in spleen and MeLN of *Apoe^−/−^*, *Apoe^−/−^ Tap1^−/−^* and *Tap1^−/−^* mice. The cells were isolated from respective tissue, stained with fluorescent antibodies and analyzed by flow cytometry. As expected, the CD8^+^ T cell fraction was depressed in the mice lacking the *Tap1* gene. Each dot in the figure represents one mouse. ***P<0.001.

**Figure 2:**
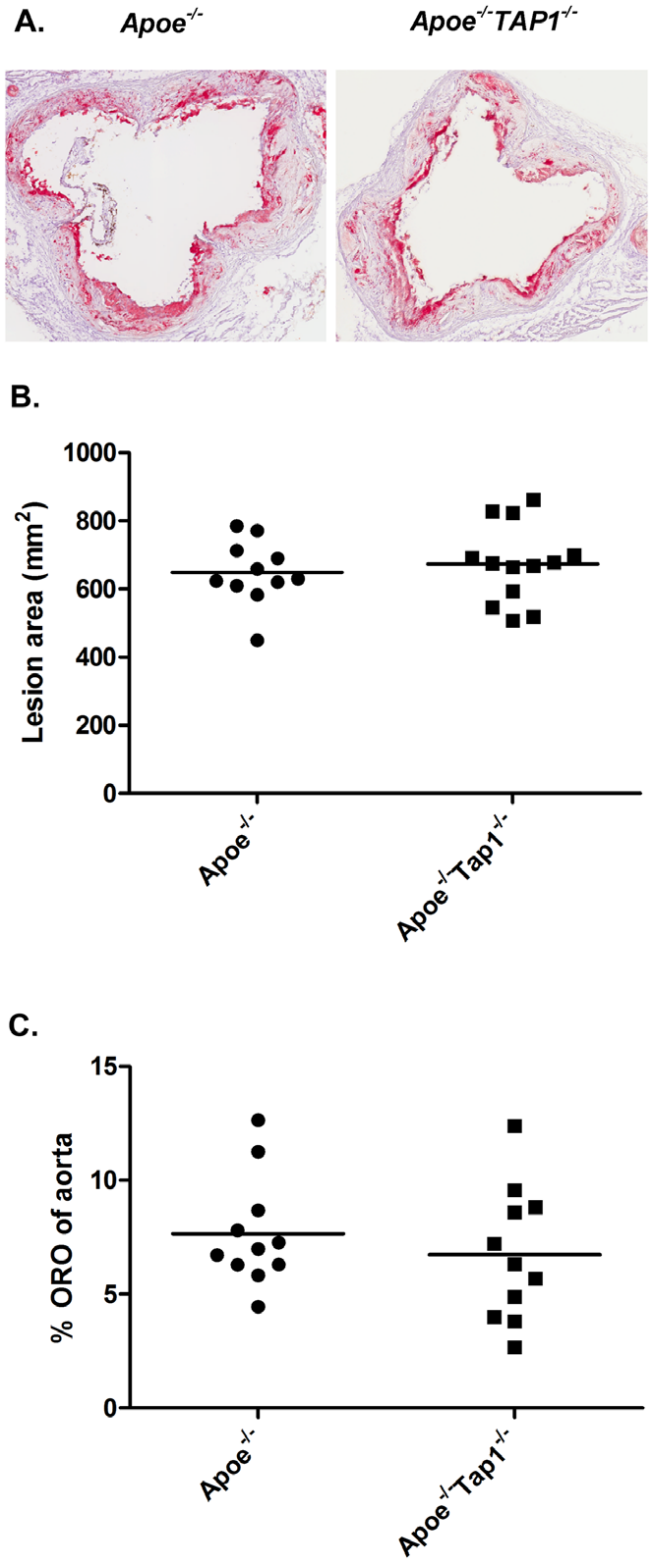
Plaque area in aortic root and aorta. (A) Representative images of plaques in the aortic root from one mouse in each group. Quantification of plaque area in the aortic root (B) and aorta (C) of *Apoe^−/−^* and *Apoe^−/−^Tap1^−/−^* mice fed high fat diet for 20 weeks. The tissues were stained with Oil red O (ORO) and analyzed by a blinded observer. Each dot in the figure represents one mouse.

We first quantified CD8^+^ T cells in *Apoe^−/−^* mice and counterparts deficient in the MHC class I transporter TAP1. As expected the CD8^+^ T cell fraction and numbers were low in *Apoe^−/−^Tap1^−/−^* and *Tap1^−/−^* mice in comparison to *Apoe^−/−^* mice, constituting ∼1% of the lymphocyte population (figure 1A–C). However, after 20 weeks of high fat diet the *Apoe^−/−^Tap1^−/−^* mice had equal lesion size compared to *Apoe^−/−^* mice in aortic root (figure 2A and 2B), aorta (figure 2C) and greater- respective lesser curvature of the aortic arch (data not shown). WT and *Tap1^−/−^* mice did not develop quantifiable lesions and were excluded from analysis. The same pattern was found in mice given high fat diet for 8 weeks (table 1). Since TAP1 deficiency could influence inflammation and lipid accumulation in the lesions we analyzed the T cell-, macrophage- and lipid content in the old mice as indicators of lesion stability. No differences were detected in lesion infiltration of macrophages (20.8±5.8% vs. 21.3±3.8%) or CD3^+^ T cells (30.7±10.2% vs. 25.6±3.6%) in *Apoe^−/−^Tap1^−/−^* compared to *Apoe^−/−^* mice. There were no detectable amounts of CD8^+^ T cells in lesions of mice given HFD for 22 weeks (data not shown). Further, no differences were found in CD4^+^ T cell lesion infiltration in *Apoe^−/−^Tap1^−/−^* compared to *Apoe^−/−^* mice (data not shown). The neutral lipid accumulation in the lesions was not affected (11.2±4.5% in *Apoe^−/−^Tap1^−/−^* vs. 13.8±4.3% in *Apoe^−/−^*). Thus, TAP1 deficiency does not induce severe alterations in the intra-lesional milieu. Systemically, plasma cholesterol and triglyceride levels were not different between groups on *Apoe^−/−^* background but as expected levels were increased in these groups compared to mice on an *Apoe^+/+^* background (table 1 and 2). The weights did not differ between *Apoe^−/−^* and *Apoe^−/−^Tap1^−/−^* mice (table 1 and 2).

### Cell characterization

**Figure 3:**
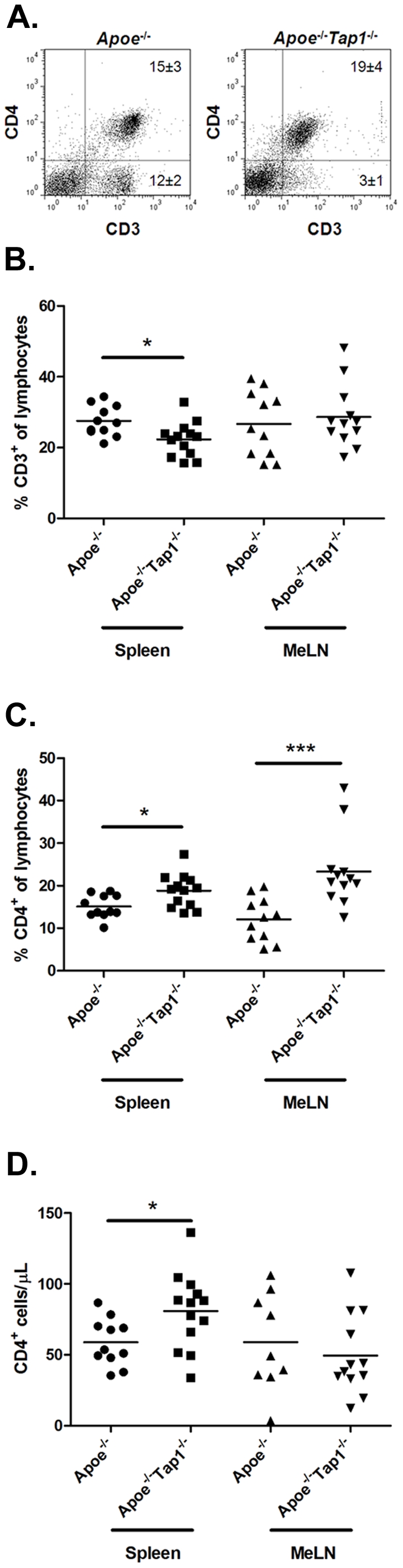
CD3^+^ T cells and CD4^+^ T cells in spleen and MeLN. Flow cytometry graphs showing the cell populations in spleen from one representative mouse from each group (A). Numbers given in the graphs are per cent cells out of all lymphocytes. The fraction of CD3^+^ T cells (B), CD3^+^CD4^+^ T cells (C) and CD3^+^CD4^+^ T cell numbers (D) in *Apoe^−/−^* and *Apoe^−/−^ Tap1^−/−^* mice in spleen and MeLN. The cells were isolated from respective tissue, stained with fluorescent antibodies and analyzed by flow cytometry. Each dot in the figure represents one mouse. *P<0.05, ***P<0.001.

**Figure 4:**
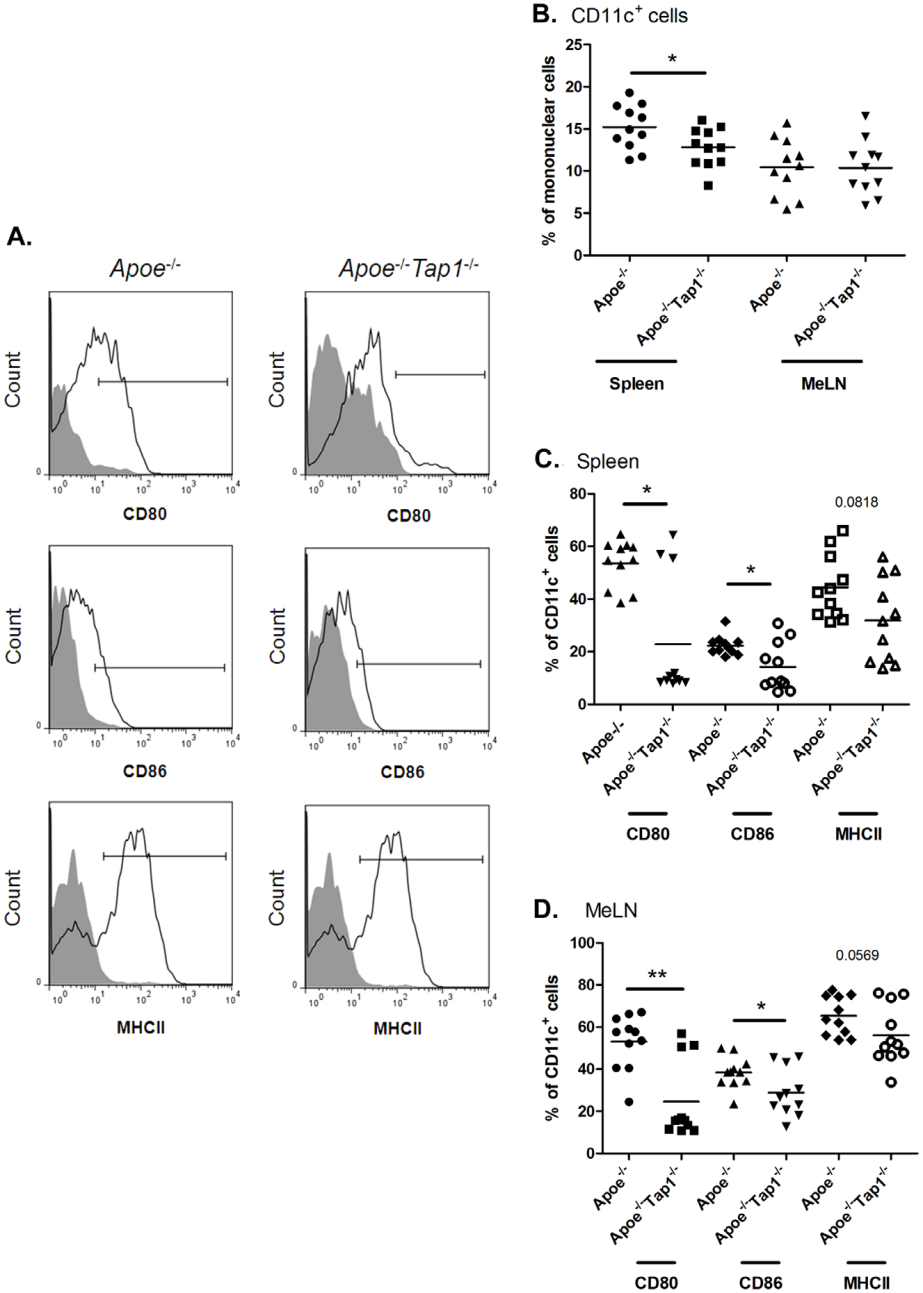
CD11c^+^ cells and expression of CD80^+^, CD86^+^ and MHC class II^+^ on CD11c^+^ cells in spleen and MeLN. Histograms corresponding to the cell populations in the spleen from one representative mouse from each group (A). Gate boundaries were set by fluorescence minus one controls (solid grey). The fraction of CD11c^+^ cells (B) and CD11c^+^CD80^+^, CD11c^+^CD86^+^ and CD11c^+^MHCII^+^ cells in spleen (C) and MeLN (D) of *Apoe^−/−^* and *Apoe^−/−^ Tap1^−/−^* mice. The cells were isolated from respective tissue, stained with fluorescent antibodies and analyzed by flow cytometry. Each dot in the figure represents one mouse. *P<0.05, **P<0.01.

The T cell and dendritic cell (DC) populations of spleen and MeLN were analyzed to characterize the response to hypercholesterolemia systemically and in conjunction to lesions. While viable CD3^+^ T cells were less abundant in spleen of *Apoe^−/−^Tap1^−/−^* mice (figure 3A and 3B), there was no difference in MeLN compared to *Apoe^−/−^* mice (figure 3B). The fraction of CD3^+^CD4^+^ T cells was higher in *Apoe^−/−^Tap1^−/−^* mice and *Tap1^−/−^* mice compared to *Apoe^−/−^*mice in both spleen and MeLN (figure 3A and 3C and data not shown). While the increase in spleen was moderate, the CD3^+^CD4^+^ T cell compartment in MeLN was almost doubled (figure 3C), which may explain the difference in CD3^+^ T cells between the groups in spleen compared to the indifference between groups in MeLN (figure 3B). However, since the number of CD4^+^ T cells did not differ in MeLN (figure 3D) the relative rise in the CD4^+^ T cell population is likely a result of the diminished CD8^+^ T cell population. Interestingly, the number of CD4^+^ T cells in spleen increased in the *Apoe^−/−^Tap1^−/−^* mice (figure 3D). Since this population could mediate inflammation we analyzed the fraction of memory effector cells. There was no difference in CD4^+^CD44^+^CD62L^−^ T cells in *Apoe^−/−^Tap1^−/−^* mice compared to *Apoe^−/−^*mice in MeLN or spleen (figure S1A). Surprisingly, the corresponding CD8^+^ population was approximately eight times larger in MeLN and spleen of *Apoe^−/−^Tap1^−/−^* mice compared to *Apoe^−/−^*mice (figure S1B). However, the CD8^+^CD44^+^CD62L^+^CD122^+^ regulatory T cell [19] population in the small CD8^+^ T cell population was also higher in *Apoe^−/−^Tap1^−/−^* mice (figure S2), which could compensate rise in effector cells. In contrast, within the CD4^+^ population the CD4^+^CD25^+^FoxP3^+^ regulatory T cell fraction was decreased in MeLN but not in spleen of *Apoe^−/−^Tap1^−/−^* mice given HFD for 8 weeks (14 weeks old at death) compared to equivalent *Apoe^−/−^* mice (figure S3). Since the major activation pathway of CD8^+^ T cells occur via DCs we analyzed abundance and activation of CD11c^+^ cells in spleen and MeLN of 26 weeks old mice. The fraction of CD11c^+^ cells in spleen, but not in MeLN, was lower in *Apoe^−/−^Tap1^−/−^* mice compared to *Apoe^−/−^*mice (figure 4B). Further, CD11c^+^ cells in both organs expressed lower levels of CD80, CD86 and a trend towards decreased MHC class II levels (figure 4A, 4C and 4D).

### Cell characterization of proliferated lymphoid cells

In order to elucidate the proliferative response of CD4^+^ and CD8^+^ T cells, lymphocyte suspensions from spleen and MeLN were incubated with ConA for two days. The fraction of respective cell type was assessed at day 0 and day 2 using flow cytometry analyses. The cell pattern at day 0 is presented in figure 1 and 3. However, incubation with ConA resulted in a robust rise in CD3^+^CD8^+^ T cells at day 2 in the *Apoe^−/−^Tap1^−/−^* mice in both spleen and MeLN (figure S4). Interestingly, the CD3^+^CD8^+^ T cell population at day 2 was also increased in *Apoe^−/−^*mice in comparison to CD3^+^CD4^+^ T cells (figure S4). This may indicate that the CD3^+^CD8^+^ T cells respond strongly to the ConA incubation while the CD3^+^CD4^+^ T cells are weak responders.

### Proliferation of splenocytes

**Figure 5:**
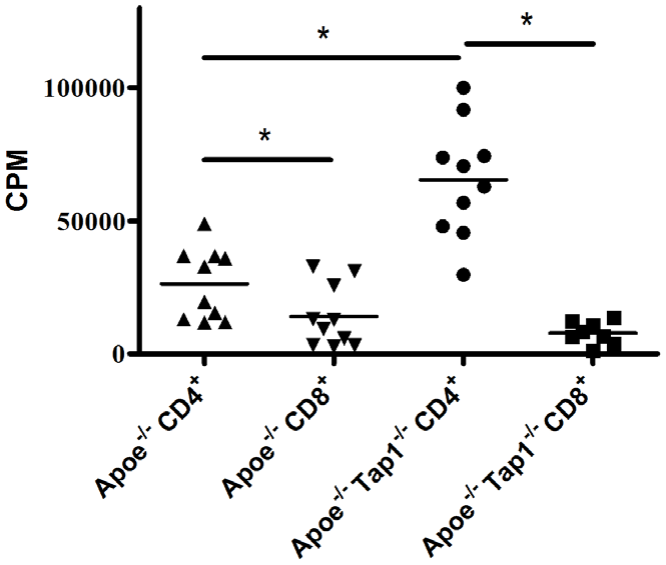
Proliferation of splenic CD4^+^ and CD8^+^ co-cultures of young mice. Proliferation were analysed in ConA stimulated (90 hrs) CD4^+^:CD11c^+^ (CD4^+^) and CD8^+^:CD11c^+^ (CD8^+^) cell co-cultures in *Apoe^−/−^* and *Apoe^−/−^Tap1^−/−^* mice with presence of radioactive labelled thymidine during the last 16–20 hrs. The cells were isolated from spleens of mice given high fat diet for 8 weeks and magnetically separated into CD11c^+^, CD4^+^ and CD8^+^ cells. CPM denotes counts per minute. Each dot in the figure represents one mouse. *P<0.05.

To test the impact of antigen presenting cells (APCs) on the proliferation of separated CD4^+^ and CD8^+^ T cells we performed analysis in mice given high fat diet for 8 weeks (14 weeks old at death). The CD4^+^ and CD8^+^ T cells from *Apoe^−/−^Tap1^−/−^* mice and *Apoe−/−* mice were magnetically separated and stimulated for three days with ConA and an equal amount of CD11c^+^ cells. In this setting the CD8^+^ T cells proliferate poorly in both *Apoe^−/−^Tap1^−/−^* mice and *Apoe−/−* mice (figure 5). In contrast, CD4^+^ T cells proliferate more than the CD8^+^ T cells in both groups. Interestingly, CD4^+^ T cells had an increased proliferation in *Apoe^−/−^Tap1^−/−^* mice compared to *Apoe^−/−^* mice (figure 5). Stimulation of T cells with ConA is considered to give an estimation of the T cell priming status from the *in vivo* environment. Thus, cells in the CD4^+^ co-culture seems to be more pre-primed compared to cells in the CD8^+^ co-culture and more in TAP deficient animals.

### Plasma cytokines

The plasma level of IL-12, IL-1β, KC, IFN-γ, TNF-α, IL-2, IL-10, IL-4 and IL-5 was analyzed but no differences between the groups of mice were found (data not shown).

## Discussion

T cell immunity plays an important role in the development of atherosclerosis, but the participation of CD8^+^ T cells and MHC class I antigen presentation has not been fully investigated. Previous reports on the role of CD8^+^ T cells in the development of atherosclerosis are few and describe a diverse impact on disease indicating a need for further characterization. We recently evaluated the CD8^+^ T cell response to hypercholesterolemia in an *Apoe^−/−^* mouse model leading to the conclusion that initial atherosclerosis is characterized by a CD8^+^ T cell response which was more rapid and stronger than the corresponding CD4^+^ T cell response [13]. The hypothesis of the current study was therefore that an *Apoe^−/−^* mouse model with few CD8^+^ T cells (*Apoe^−/−^Tap1^−/−^*) would have a decreased lesion development in response to a high fat diet compared to an *Apoe^−/−^* mouse model with normal levels of CD8^+^ T cells. However, lesion size was found to be equal in the *Apoe^−/−^Tap1^−/−^* mice and *Apoe^−/−^* mice. As lesion size in younger mice display the same pattern, the lesion severity may be of less importance in this system. However, as the diet composition may impact the presence of antigens implicated in autoimmune lymphocyte response, experimental groups given chow diet could have been included in the study. Interestingly, a study in which chow-fed *Apoe^−/−^* mice with a mutation in the CD8-encoding gene Lyt-2 developed equivalent atherosclerosis as *Apoe^−/−^* mice with functional CD8^+^ T cells [7]. Accordingly, neither loss of function in CD8^+^ T cells nor the TAP1-dependent CD8^+^ T cell activation is essentially affecting the lesion development. The result raises the question if 1) the CD4^+^ T cell population compensate pro-inflammatory effects of the normal CD8^+^ T cell population or 2) the CD8^+^ T cell population is not involved in the disease process. The CD4^+^ fraction of *Tap1^−/−^* and *Apoe^−/−^Tap1^−/−^* mice was enlarged compared to TAP1-expressing mice in contrast to studies in *Tap1^−/−^* mice [14] and *Tap1^−/−^* humans [20] having normal CD4^+^ levels. Although the effector memory CD4^+^ T cell population was equal in both groups, the actual number of cells was larger in *Apoe^−/−^Tap1^−/−^* mice due to the enlarged CD4^+^ T cell population. Moreover, the CD4^+^CD25^+^FoxP3^+^ regulatory T cell population was decreased in MeLN of the group given HFD for 8 weeks supporting a disease-driving property of this T cell subset. Hence, pro-atherogenic cells within this population may maintain disease by compensating for the loss of CD8^+^ T cells. Apart from this, diminishment of the CD8^+^ T cell population may reduce the impact of both pro- and anti-inflammatory CD8^+^ T cells on atherosclerosis. This could be a transient process or affect either of the cell populations more than the other. Interestingly, incubation of lymphocytes with ConA resulted in an increased fraction of CD8^+^ T cells while the CD4^+^ T cells were weak responders and displayed a decrease in the fraction of lymphocytes. This supports the data presented in our previous study [13] and results by Aldrich *et al.* describing a strong antigen-specific response in CD8^+^ T cells from *Tap1^−/−^*mice [21]. However, when splenic CD4^+^ or CD8^+^ T cells were mixed with CD11c^+^ cells and stimulated with the polyclonal T cell activator ConA, there was an increased proliferation in CD4^+^ cultures from *Apoe^−/−^Tap1^−/−^* mice compared to corresponding CD8^+^ cultures. Furthermore, the CD4^+^ cultures from *Apoe^−/−^Tap1^−/−^* mice proliferated more than corresponding cultures in *Apoe^−/−^* mice. Since CD8^+^ T cells benefit from CD4^+^ T cell assistance [22] the proliferation of the isolated CD8^+^ T cells may be negatively regulated. In contrast, the CD4^+^ T cells in the *Apoe^−/−^Tap1^−/−^* mice seem to be more pre-primed than in the *Apoe−/−* mice. Taken together, the data may indicate that CD8^+^ T cells need CD4^+^ T cell help to proliferate properly and CD4^+^ T cell proliferation may be inhibited by CD8^+^ T cell proliferation in a co-culture. Since the results of this *in vitro* testing seem to have little impact on the disease mechanism it may not be reflected *in vivo*. The *in vivo* system may promote other functional mechanisms and/or the CD8^+^ T cells may not respond to endogenous antigens present in the *in vivo* system as strong as they respond to ConA. Thus, the CD8^+^ T cell population in *Apoe^−/−^Tap1^−/−^* mice was only 12% of that in *Apoe^−/−^* mice but it contained relatively more effector memory- and regulatory T cells. Although CD8^+^ T cells in TAP1-deficient mice are known to be functional [23], the effector cell- and regulatory T cell population here was still smaller than in *Apoe^−/−^* mice, giving them a limited impact on atherosclerosis.

Since about one third of all MHC class I-peptide complexes are presented via the TAP1-independent pathway [24] it was expected that abundance as well as activation of CD11c^+^ DCs would be lower in *Apoe^−/−^Tap1^−/−^* mice than *Apoe^−/−^* mice as was demonstrated in the present study. Considering the size of the CD8^+^ T cell population the remaining CD11c^+^ cell population may be sufficient to increase the CD8^+^ effector memory cell population. Consequently, the larger CD4^+^ T cell population may not be adequately stimulated to achieve a similar rise in effector memory cells. However, activation of CD4^+^ and CD8^+^ T cells involve not only APC derived MHC class I/II-TCR contact but also stimulation from CD4^+^ T cells. They can acquire MHC class I/II-peptide complexes from APCs and together with co-stimulatory molecules activate CD4^+^ or CD8^+^ T cells and increase the APC derived stimulation [22]. Thus, the decreased DC activation in *Apoe^−/−^Tap1^−/−^* mice may be compensated by antigen presentation mediated by the increased CD4^+^ T cell population, potentially activating both pro-inflammatory CD4^+^ and CD8^+^ T cell subsets. However, activation of CD4^+^ and/or CD8^+^ T cell subsets targeting antigens present in atherosclerotic lesions would result in increased lesional T cell infiltration. As the amount of T cell subsets was similar or below detection limit the impact of T cells for lesion development may be questioned in this model. Moreover, the macrophage lesion infiltration was not affected indicating that TAP1 deficiency does not exert major APC-driven effects on atherosclerosis progression. Thus, the similar lesion size in *Apoe^−/−^* mice and *Apoe^−/−^Tap1^−/−^* mice reflects a minor role of TAP1 for atherosclerosis development. However, the mice of this study were housed in an environment with low exposure to infections. Several pathogens, including *Chlamydia pneumoniae*, herpes simplex virus and cytomegalovirus have been associated with atherosclerosis [25]. As CD8^+^ T cells are activated by bacterial/viral antigens via TAP dependent- and independent pathways [16] it is conceivable that the model of this study contains limitations. Thus, assuming a low infectious burden in the mice of the present study, a reduced amount of autoreactive CD8^+^ T cells targeting lesion specific antigens may favor other atherogenic cell types.

In conclusion, hypercholesterolemic *Apoe^−/−^* mice that lack TAP1-dependent antigen presentation develop atherosclerotic lesions, inflammatory cell infiltration and lipid accumulation equal to *Apoe^−/−^* mice. The relative CD4^+^ T cell population was larger in both local and systemic lymphoid tissues in *Apoe^−/−^Tap1^−/−^* mice compared to *Apoe^−/−^* mice, whereas CD4^+^ T cell numbers were increased in spleen but not in MeLN. In addition, the CD11c^+^ population had a lower expression of activation markers in *Apoe^−/−^Tap1^−/−^* mice. Although the effector memory T cell population was larger in the small CD8^+^ T cell population of *Apoe^−/−^Tap1^−/−^* mice the impact on disease is likely to be limited. Taken together, the present study provides novel information indicating a limited role of TAP1 and CD8^+^ T cells in atherosclerosis development.

## Supporting Information

Figure S1 **Effector memory CD4^+^ T cells and CD8^+^ T cells in spleen and MeLN.** The fraction of (A) CD3^+^CD4^+^CD44^+^CD62L^−^ and (B) CD3^+^CD8^+^ CD44^+^CD62L^−^ T cells in spleen and MeLN of *Apoe^−/−^* and *Apoe^−/−^ Tap1^−/−^* mice. The cells were isolated from respective tissue, stained with fluorescent antibodies and analyzed by flow cytometry. Each dot in the figure represents one mouse. ***P<0.001.
(TIF)

Figure S2 **Regulatory CD8^+^ T cells in spleen and MeLN.** Analysis of CD3^+^CD8^+^CD44^+^CD62L^+^ CD122^+^ T cells in *Apoe^−/−^* and *Apoe^−/−^ Tap1^−/−^* mice in spleen and MeLN. The cells were isolated from respective tissue, stained with fluorescent antibodies and analyzed by flow cytometry. Each dot in the figure represents one mouse. ** *P*<0.01, ***P<0.001.
(TIF)

Figure S3 **Regulatory CD4^+^ T cells in spleen and MeLN of young mice.** Analysis of CD3^+^ CD4^+^CD25^+^FoxP3^+^ T cells in spleen and MeLN of *Apoe^−/−^* and *Apoe^−/−^ Tap1^−/−^* mice given HFD for 8 weeks. The cells were isolated from respective tissue, stained with fluorescent antibodies and analyzed by flow cytometry. Each dot in the figure represents one mouse. **P<0.01.
(TIF)

Figure S4 **ConA-stimulation of lymphocytes.** Lymphocytes from spleen and MeLN were incubated with ConA for 2 days and the relative fraction of CD4^+^ and CD8^+^ T cells was assessed by flow cytometry analysis. The CD8^+^ T cell fraction was larger than the CD4^+^ T cell fraction in spleen and MeLN of both *Apoe^−/−^* and *Apoe^−/−^ Tap1^−/−^* mice. ** *P*<0.01, ***P<0.001.
(TIF)
