# Supplementary figures and images for: TAP1-Deficiency Does Not Alter Atherosclerosis Development in Apoe −/− Mice
Source: PLoS One. 2012 Mar 30;7(3):e33932. doi: 10.1371/journal.pone.0033932 (PMC3316507; doi:10.1371/journal.pone.0033932)

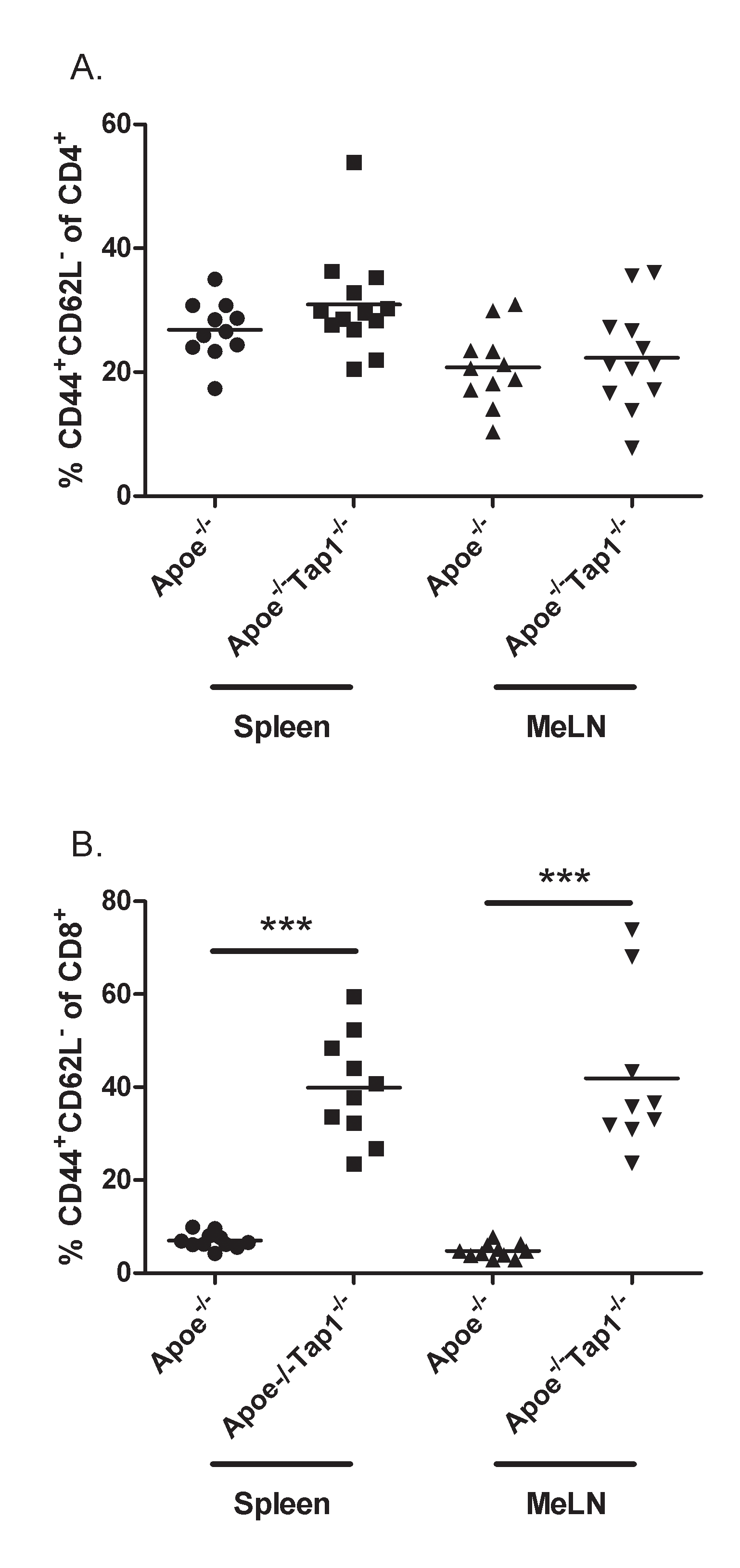

Supplement: Figure S1 — Effector memory CD4+ T cells and CD8+ T cells in spleen and MeLN. The fraction of (A) CD3+CD4+CD44+CD62L− and (B) CD3+CD8+ CD44+CD62L− T cells in spleen and MeLN of Apoe−/− and Apoe−/− Tap1−/− mice. The cells were isolated from respective tissue, stained with fluorescent antibodies and analyzed by flow cytometry. Each dot in the figure represents one mouse. ***P<0.001. (TIF) [file pone.0033932.s001.tif]

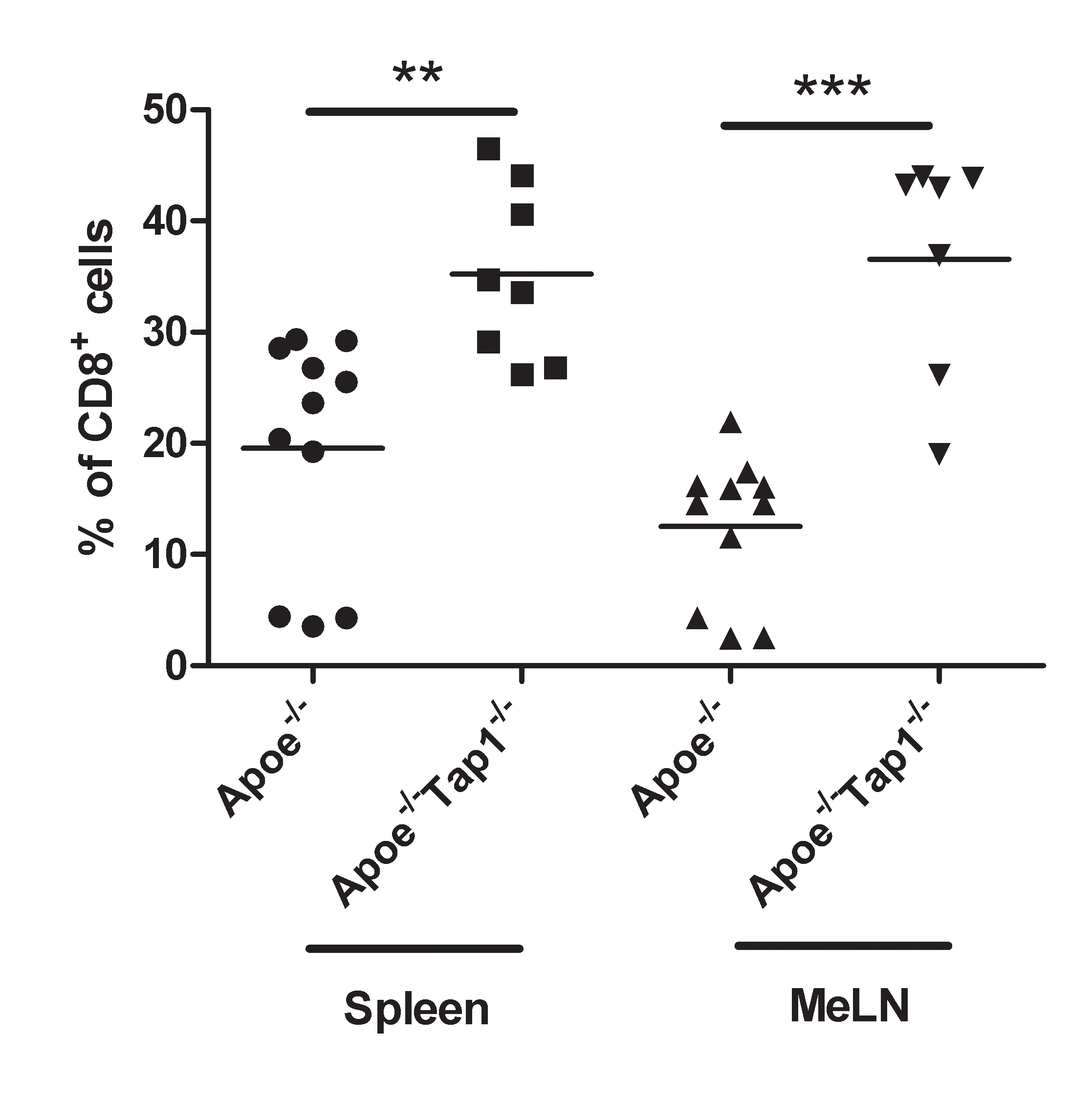

Supplement: Figure S2 — Regulatory CD8+ T cells in spleen and MeLN. Analysis of CD3+CD8+CD44+CD62L+ CD122+ T cells in Apoe−/− and Apoe−/− Tap1−/− mice in spleen and MeLN. The cells were isolated from respective tissue, stained with fluorescent antibodies and analyzed by flow cytometry. Each dot in the figure represents one mouse. ** P<0.01, ***P<0.001. (TIF) [file pone.0033932.s002.tif]

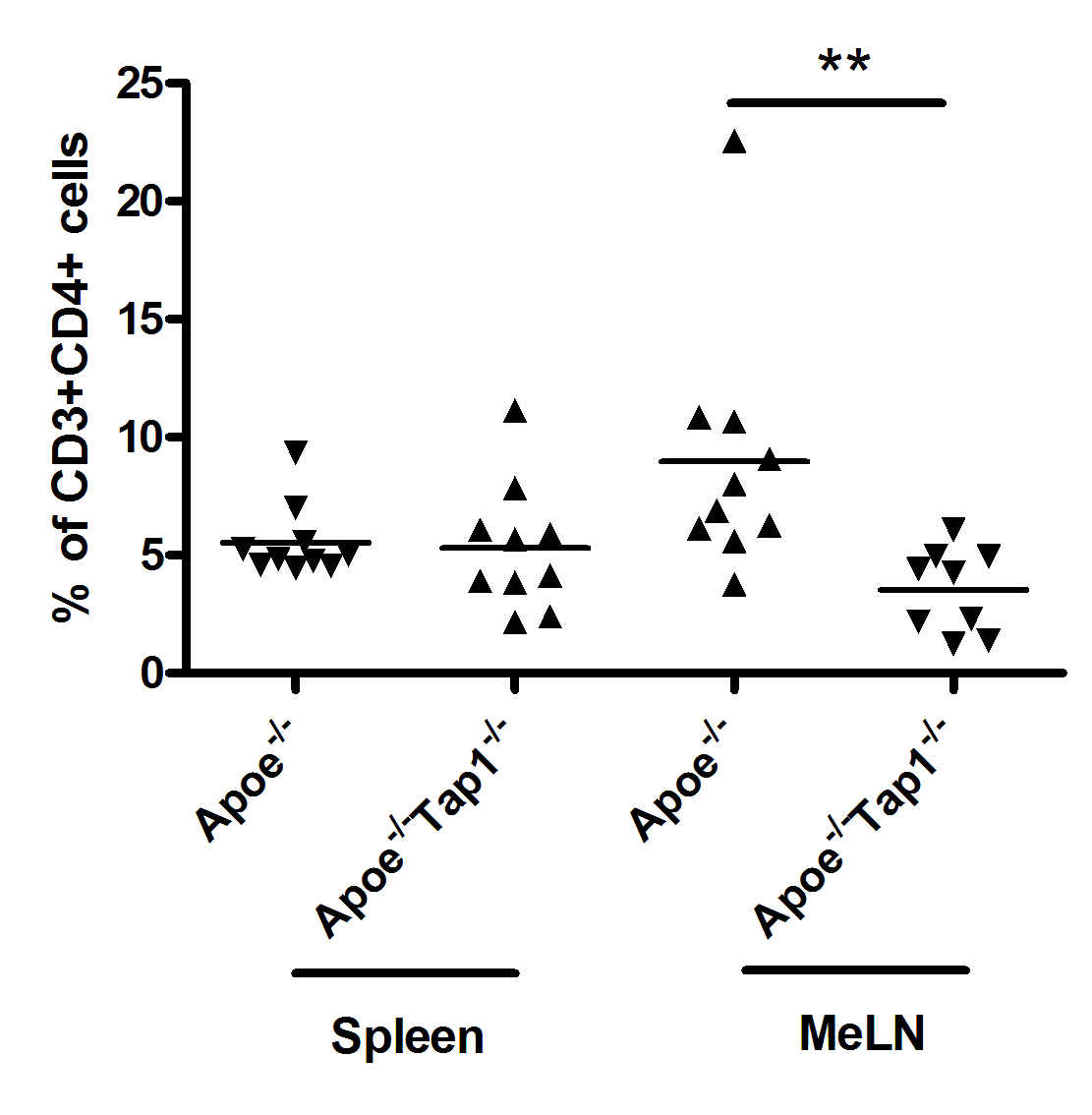

Supplement: Figure S3 — Regulatory CD4+ T cells in spleen and MeLN of young mice. Analysis of CD3+ CD4+CD25+FoxP3+ T cells in spleen and MeLN of Apoe−/− and Apoe−/− Tap1−/− mice given HFD for 8 weeks. The cells were isolated from respective tissue, stained with fluorescent antibodies and analyzed by flow cytometry. Each dot in the figure represents one mouse. **P<0.01. (TIF) [file pone.0033932.s003.tif]

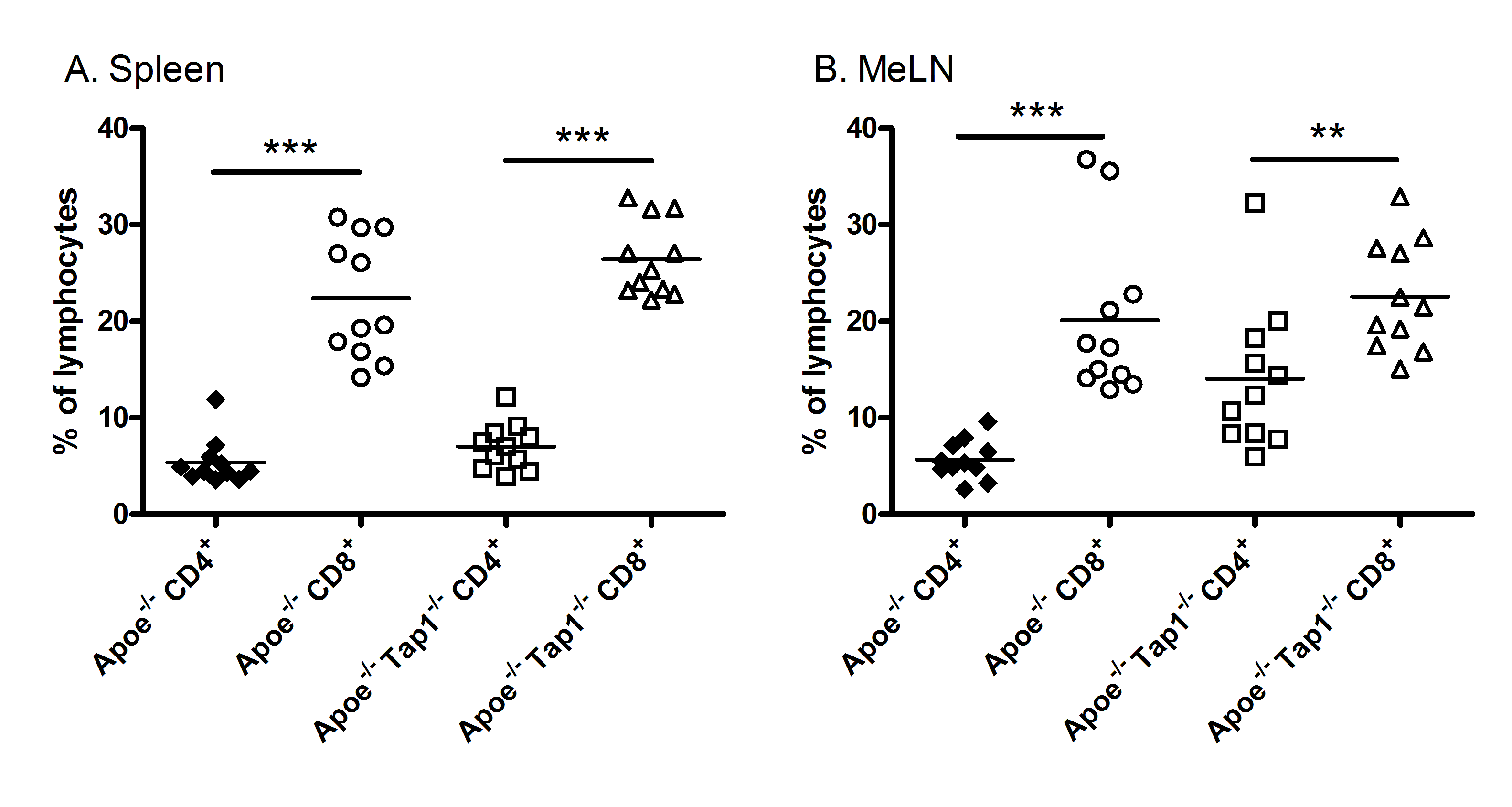

Supplement: Figure S4 — ConA-stimulation of lymphocytes. Lymphocytes from spleen and MeLN were incubated with ConA for 2 days and the relative fraction of CD4+ and CD8+ T cells was assessed by flow cytometry analysis. The CD8+ T cell fraction was larger than the CD4+ T cell fraction in spleen and MeLN of both Apoe−/− and Apoe−/− Tap1−/− mice. ** P<0.01, ***P<0.001. (TIF) [file pone.0033932.s004.tif]
